# Macrophage-associated noncoding RNAs in atherosclerosis and chronic kidney disease

**DOI:** 10.3389/fcvm.2026.1884576

**Published:** 2026-08-04

**Authors:** Prabhash Kumar Jha, Caio Borges Nascimento, Adrien Lupieri, Elena Aikawa, Masanori Aikawa

**Affiliations:** 1 Center for Excellence in Vascular Biology, Brigham and Women’s Hospital, Harvard Medical School, Boston, MA, United States; 2 Center for Interdisciplinary Cardiovascular Sciences, Brigham and Women’s Hospital, Harvard Medical School, Boston, MA, United States; 3 Channing Division of Network Medicine, Brigham and Women’s Hospital, Harvard Medical School, Boston, MA, United States

**Keywords:** atherosclerosis, cardio-kidney-metabolic disease, cardiovascular disease, chronic kidney disease, inflammation, long noncoding RNA, macrophage activation, macrophage function

## Abstract

Although approximately 97% of the human genome does not encode proteins, it produces a vast repertoire of regulatory RNA transcripts that play critical roles in cellular and molecular processes. Among these, noncoding RNAs including microRNAs and long noncoding RNAs have emerged as key regulators of gene expression through their interactions with DNA, RNA, and proteins. Increasing evidence indicates that noncoding RNAs are central to immune regulation and inflammatory signaling, positioning them as important contributors to complex chronic diseases. Chronic kidney disease (CKD) is of particular interest because it represents a growing global health burden with strong inflammatory and metabolic overlap with cardiovascular disease, placing it at the center of cardio-kidney-metabolic disease. In both atherosclerosis and CKD, a pro-inflammatory milieu characterized by sustained macrophage accumulation and activation drives vascular dysfunction, organ ischemia, and progressive tissue injury. Macrophages act as pivotal instigators of these cardio-kidney-metabolic pathologies by integrating inflammatory signaling, lipid handling, and tissue remodeling processes. This review discusses current knowledge on the roles of noncoding RNAs in regulating macrophage function in atherosclerosis and CKD, with particular emphasis on the molecular mechanisms underlying inflammatory responses and disease progression. By highlighting shared and disease-specific noncoding RNA–mediated pathways, this review aims to provide insight into novel regulatory networks and potential therapeutic targets in cardio-kidney-metabolic disease.

## 1 Introduction

Initially labeled as “junk DNA,” the noncoding portion of the human genome comprises approximately 76%–99% of the total genetic sequence, with most sources estimating at least 95% (1–3). This portion gives rise to a diverse array of transcripts collectively termed noncoding RNAs, which are broadly classified according to transcript length. Transcripts longer than 200 nucleotides are defined as long noncoding RNAs (lncRNAs), whereas shorter transcripts are classified as small noncoding RNAs (sncRNAs) (4). These two major subtypes are further subdivided into multiple classes based on structural features and biological functions (5).

LnRNAs represent a highly diverse class of transcripts with a wide range of regulatory functions. Integrative annotation efforts, such as LncBook 2.0, report approximately 95,000 putative human lncRNA genes and more than 320,000 transcript isoforms, highlighting the extensive transcriptional complexity of the noncoding genome (6). LncRNAs can originate from most genomic regions, may be transcribed in sense or antisense orientation, and can even share genomic loci with protein-coding genes through alternative splicing events (7–9). They regulate gene expression and cellular function through diverse mechanisms involving interactions with proteins, DNA, and other RNAs, both coding and noncoding (10, 11). Despite increasing functional annotation, the vast majority of identified lncRNAs remain poorly characterized; nevertheless, emerging literature enables them to be broadly categorized based on common regulatory themes.

Compared with protein-coding RNAs, lncRNAs generally exhibit lower sequence conservation and expression levels, higher cellular and tissue specificity, and more restricted spatiotemporal expression patterns (12, 13). They frequently associate with cellular development and are often transiently expressed during growth and differentiation processes (14). Accordingly, lncRNAs contribute to the regulation of complex cellular programs essential for cell specialization in higher organisms. This concept is supported by the strong correlation between organismal complexity and the extent of transcribed noncoding RNA within the genome, a relationship considerably stronger than that observed between complexity and the number of protein-coding genes (15). Moreover, lncRNAs can exert direct transcriptional effects in response to specific environmental stimuli in both plants and animals (16–18). In this context, it is plausible that lncRNAs play important roles in regulating differentiation and heterogeneity of macrophages in the pathogenesis of inflammatory cardio-kidney-metabolic diseases (19–22). LncRNAs may also contribute to other pathological macrophage behaviors observed in these conditions, including enhanced monocyte chemotaxis (23) and foam cell formation (24) and impaired efferocytosis.

Among small noncoding RNAs, microRNAs (miRNAs), which are typically 21–25 nucleotides in length, are the most extensively studied subtype. More than 38,000 miRNAs have been identified and cataloged in public databases, making them the best-characterized class of noncoding RNAs to date (25). Their canonical mechanism of action involves guiding the RNA-induced silencing complex (RISC) to complementary sequences within the untranslated regions (UTRs) of target messenger RNAs (mRNAs), resulting in transcript degradation or translational repression (26). miRNA function, however, is not limited to gene silencing. In certain contexts, miRNAs can promote translation of target mRNAs (27) and may also act as intercellular signaling molecules by binding to Toll-like receptors (TLRs) (28). Consequently, miRNAs can function as both repressors and activators of gene expression and may exert context-dependent effects at different stages of the cell cycle (28). Indeed, several studies suggest that miRNAs collectively regulate the majority of the human transcriptome and influence all fundamental stages of cellular development, as well as numerous tissue-specific processes required for homeostasis (29). Consistent with this broad regulatory role, genetic disruption of key miRNA biogenesis components in mice results in embryonic lethality (30) or severe organ dysfunction, including hypertrophic cardiomyopathy (31). It is therefore consistent with current knowledge that miRNAs play important roles in abnormal cellular behaviors and homeostatic imbalances commonly observed in cardio-kidney-metabolic disorders, including arterial plaque formation, excessive pro-inflammatory signaling, and enhanced fibrotic responses (32).

Building on these observations, this review examines how noncoding RNAs regulate macrophage function in atherosclerosis and CKD within the broader cardio-kidney-metabolic disease framework, where closely interconnected cardiac and renal pathways contribute to disease progression across multiple organ systems (33). Of particular relevance to this review, chronic kidney disease (CKD) drives metabolic disturbances and a persistent systemic pro-inflammatory state that jointly accelerate cardiovascular dysfunction, positioning CKD as a central driver of cardio-kidney-metabolic diseases (34–36). Patients with CKD exhibit a 10- to 20-fold higher risk of cardiovascular mortality compared with age- and sex-matched individuals without CKD (37), an association that remains significant even after adjustment for traditional risk factors such as diabetes and hypertension (38). Macrophage activation and recruitment are central features of this pro-inflammatory state (19, 39) and are known to be regulated by multiple noncoding RNAs.

This review focuses on the roles of noncoding RNAs in regulating macrophage heterogeneity and function in atherosclerosis and CKD, highlighting shared and disease-specific mechanisms with therapeutic relevance.

## Macrophage functional heterogeneity in atherosclerosis and CKD

Macrophages are considered a first line of defense of the immune system against pathogens and play a critical role in inflammatory diseases (40). Macrophage heterogeneity in atherosclerosis extends far beyond the traditional M1/M2 paradigm and is now recognized as a central determinant of plaque development, stability, and therapeutic response (41–44). Single-cell and systems-level profiling have revealed a spectrum of macrophage subpopulations within lesions, including pro-inflammatory, lipid-handling, efferocytotic, and reparative phenotypes that coexist and dynamically interconvert in response to microenvironmental cues. Pro-inflammatory macrophages promote cytokine production, and necrotic core expansion, whereas efferocytotic and reparative subsets support debris clearance and tissue remodeling. Recent studies emphasize that macrophage heterogeneity is not merely descriptive but functionally instructive, shaping disease trajectory and influencing how plaques respond to therapies. Macrophage diversity exceeds simplistic polarization models and that distinct subsets differentially regulate plaque progression, regression, and remodeling. Together, these studies position macrophage heterogeneity as a driver of atherosclerotic complexity and a ke*y* axis for precision intervention (43–46).

In CKD, macrophage heterogeneity is equally complex and critically shapes both local renal pathology and systemic cardiovascular risk. The kidney contains resident macrophages with homeostatic functions as well as recruited monocyte-derived populations that expand during injury (47). In CKD, these populations diversify into phenotypically and functionally distinct subsets that can either promote tissue repair or perpetuate inflammation and fibrosis. Pro-inflammatory and pro-fibrotic macrophages drive persistent cytokine signaling, fibroblast activation, and extracellular matrix deposition, accelerating tubulointerstitial scarring and functional decline, whereas other subsets support epithelial repair and resolution (48). Importantly, this imbalance toward maladaptive macrophage states in CKD not only sustains renal injury but also contributes to systemic inflammation and heightened atherosclerotic risk (49). Thus, macrophage heterogeneity provides a mechanistic bridge linking CKD progression with cardiovascular disease (50–53).

Together, these observations indicate that macrophage heterogeneity is a key determinant of disease progression in both atherosclerosis and CKD. Recent technological advances are further transforming the study of macrophage biology in cardiovascular and renal diseases. Single-cell and spatial transcriptomic approaches now enable high-resolution characterization of macrophage heterogeneity within atherosclerotic plaques and diseased kidneys, revealing previously unrecognized subsets and regulatory networks associated with disease progression (43, 46, 53). In parallel, extracellular vesicle (EV)-associated noncoding RNAs have emerged as important mediators of intercellular communication, facilitating the transfer of miRNAs and lncRNAs between immune, vascular, and renal cells (54, 55) (a, b). EV-associated ncRNAs can modulate inflammatory signaling, fibrosis, and tissue remodeling, and are increasingly being investigated as biomarkers and therapeutic targets in cardio-kidney-metabolic diseases (56, 57). Together, these emerging technologies provide new opportunities to define disease-relevant macrophage states and ncRNA-mediated regulatory pathways with unprecedented resolution.

**Figure 1:**
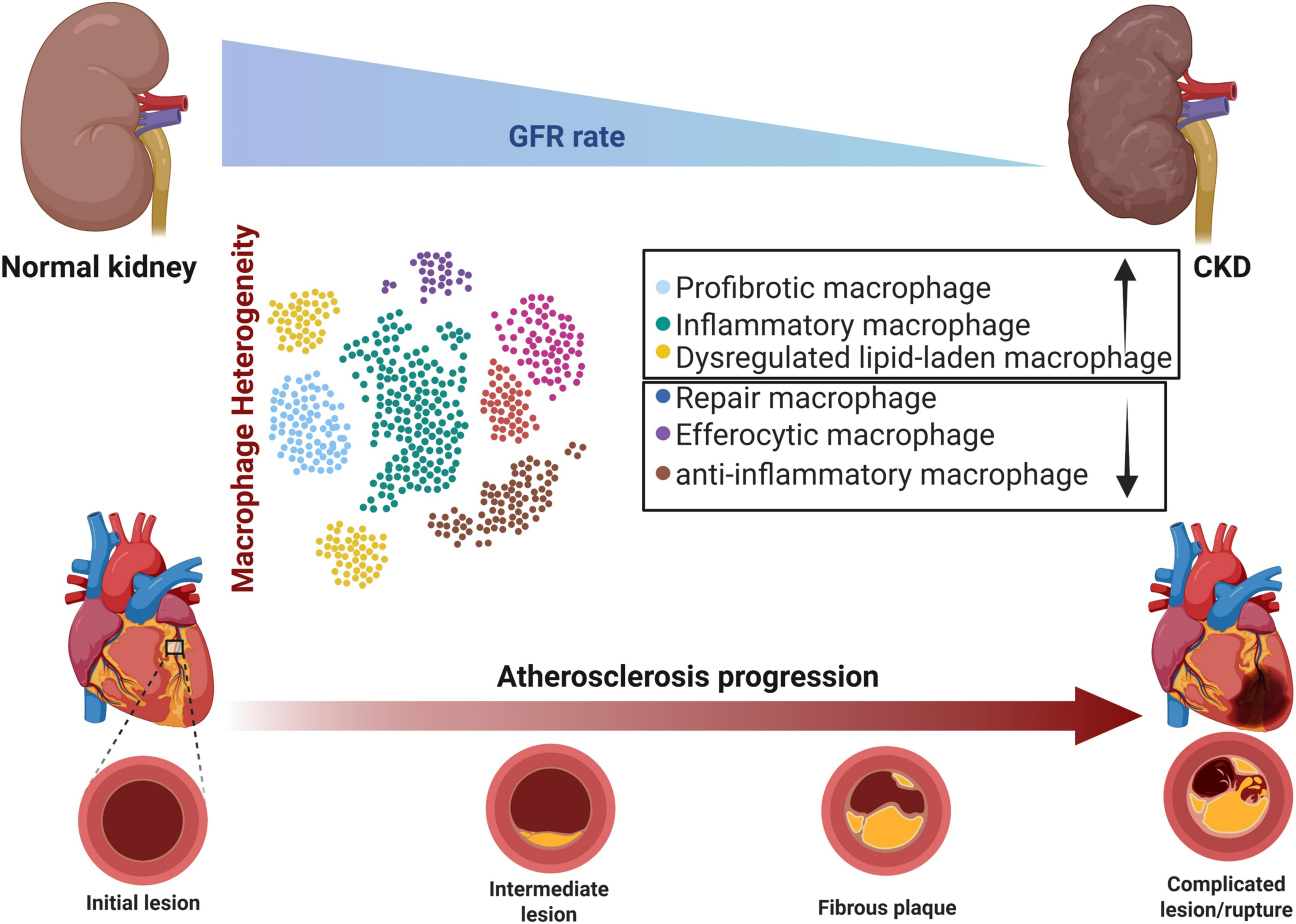
Macrophage heterogeneity as a shared, disease-specific driver of CKD and atherosclerosis. Schematic depicting macrophage heterogeneity as a common mechanistic framework across two distinct pathologies. The upper panel illustrates progressive loss of renal function from normal kidney to CKD, accompanied by a shift in macrophage populations toward profibrotic, inflammatory, and dysregulated lipid-laden macrophages, with a concomitant reduction in reparative, efferocytic, and anti-inflammatory macrophages. This imbalance promotes renal fibrosis and tissue injury. The lower panel shows the progression of atherosclerosis from initial lesion to complicated/ruptured plaque, where distinct macrophage subsets independently drive lipid accumulation, inflammation, defective efferocytosis, and plaque instability. Importantly, the figure emphasizes that while CKD and CVD are shown as separate disease processes, macrophage heterogeneity represents the shared biological principle underlying pathology in both settings, rather than a direct causal linkage between the two.

At the molecular level, this functional and phenotypic diversity is orchestrated by tightly regulated transcriptional and post-transcriptional networks, in which noncoding RNAs, particularly lncRNAs and miRNAs, have emerged as key determinants of macrophage activation programs across disease contexts. Figure 1 demonstrates macrophage heterogeneity as a shared, disease-specific driver of CKD and atherosclerosis.

## Long noncoding RNAs in macrophage activation

The ability of macrophages to occupy diverse, context-dependent activation states is controlled by layered transcriptional and post-transcriptional programs. Among these, lncRNAs have emerged as critical regulators that integrate environmental cues with gene expression networks to shape inflammatory and reparative macrophage functions. A previous study identified approximately 326 lncRNAs that exhibit significant differential expression across distinct macrophage activation states, including non-stimulated cells and macrophages engaged in pro-inflammatory or immunoregulatory programs (58). In a separate study, several of these lncRNAs increased in lipopolysaccharide (LPS)–treated macrophages adopting a pro-inflammatory transcriptional program compared with unstimulated cells. These transcripts were termed human long noncoding inflammation-associated RNAs (hLinfRNAs) and were ranked according to the magnitude of their induction following LPS stimulation, with hLinfRNA1 (ADORA2A-AS1) showing the highest upregulation and hLinfRNA5 the lowest (59). Among these hLinfRNAs, all but hLinfRNA3 exhibited suppressed expression upon treatment with the NF-*κ*B inhibitor SC51452, and notably, hLinfRNA1 expression decreased to levels comparable to basal expression observed prior to LPS stimulation (59). Together, these findings reinforce the central role of NF-*κ*B signaling in driving pro-inflammatory macrophage programs and directly link inflammatory transcriptional states to lncRNA regulation.

Additional lncRNAs critically involved in macrophage state-specific signaling pathways include FENDRR and LINC01010. FENDRR is a 3,099-base-pair lncRNA originally characterized for its tumor-suppressive functions; however, emerging evidence indicates that it also plays a key role in promoting pro-inflammatory macrophage activation. FENDRR expression is approximately 80-fold higher in macrophages exhibiting a pro-inflammatory program compared with those in immunoregulatory states (60). Exposure to IFN-*γ* markedly increases FENDRR expression, and THP-1 PMA-differentiated macrophages infected with FENDRR-expressing lentivirus display elevated mRNA levels of pro-inflammatory markers, accompanied by increased secretion of TNF-*α* and IL-6, without concomitant induction of immunoregulatory markers. Conversely, FENDRR knockdown suppresses pro-inflammatory gene expression, supporting its role in reinforcing inflammatory macrophage programs. Bioinformatic analyses have identified STAT3 binding sites within the FENDRR promoter, and the strong correlation between FENDRR expression and IFN-*γ*–induced STAT1 phosphorylation suggests that FENDRR-mediated enhancement of inflammatory activation occurs via a STAT1-dependent pathway (60). Although the precise downstream mechanisms remain unclear, previously reported interactions between FENDRR and multiple miRNAs in neoplastic cells raise the possibility that FENDRR functions as a molecular miRNA sponge, highlighting regulatory crosstalk among noncoding RNA classes (61, 62).

LINC01010, in contrast, is a myeloid-specific lncRNA that is upregulated in macrophages across both inflammatory and immunoregulatory programs compared with monocytes and is further induced following LPS exposure (63). siRNA-mediated silencing of LINC01010 results in reduced expression of the pan-macrophage marker CD68 and the immunoregulatory-associated marker CD206 and significantly impairs Escherichia coli phagocytosis across macrophage states, with a more pronounced effect observed in cells engaged in reparative programs. Additional experiments assessing ovalbumin uptake demonstrated that LINC01010 is required for efficient antigen processing, linking this lncRNA to core macrophage immune functions. Furthermore, siRNA-mediated silencing of LINC01010 led to significantly reduced the expression of multiple genes within the NF-*κ*B signaling pathway (63).

Finally, the lncRNA TCONS_00019715 is located in close genomic proximity to the protein-coding gene PAK1, a key regulator of cytoskeletal remodeling and cell motility in the mononuclear phagocyte system (64, 65). Knockdown of TCONS_00019715 reduces PAK1 mRNA expression, leading to decreased expression of pro-inflammatory markers and a concomitant increase in markers associated with immunoregulatory and reparative macrophage programs (64). These data suggest that TCONS_00019715 indirectly shapes macrophage activation states by modulating cytoskeletal and signaling pathways (58, 66).

## MicroRNAs in macrophage activation

In parallel with lncRNAs, miRNAs provide a rapid and highly dynamic layer of post-transcriptional control over macrophage gene expression. By fine-tuning key transcription factors and signaling pathways, miRNAs shape the balance between pro-inflammatory and anti-inflammatory programs that underlie macrophage heterogeneity in CKD and atherosclerosis. miRNAs also play central roles in regulating macrophage activation programs. Recent study shows that approximately 131 miRNAs exhibit altered expression between macrophages engaged in pro-inflammatory programs and undifferentiated macrophages, whereas around 30 miRNAs differ between undifferentiated cells and macrophages adopting immunoregulatory states (64). In addition, while some miRNAs differentiate inflammatory from reparative macrophage programs, others selectively distinguish IL-4 and IL-10–driven macrophage polarization states, reflecting functional heterogeneity within anti-inflammatory macrophages (67). These observations indicate that miRNAs contribute not only to broad macrophage activation programs but also to finer functional subclassification, likely underlying the spectrum of macrophage phenotypes observed *in vivo*.

In macrophages with a pro-inflammatory transcriptional program, several miRNAs interact with key regulators of inflammatory differentiation, including interferon regulatory factor-1 (IRF1), IRF5, and signal transducer and activator of transcription-1 (STAT1), all of which integrate into NF-*κ*B–dependent signaling networks (68, 69). miRNA-23 targets IRF1, attenuating inflammatory macrophage activation and reducing inflammation-associated processes in kidney disease (70). MiRNA-146 targets IRF5 and similarly suppresses inflammatory differentiation (71). In contrast, miRNA-155 promotes pro-inflammatory activation, in part through modulation of the SOCS1/STAT1 signaling axis (69).

MiRNA-155 is among the most extensively studied noncoding RNAs and has been implicated in regulating foam-cell activity, atherosclerotic plaque burden, and diverse inflammatory diseases (72, 73). It promotes inflammatory responses and is directly induced by classical inflammatory cues such as interferon-*γ* (IFN-*γ*). Moreover, miRNA-155 inhibits B-cell lymphoma-6 (BCL6), a negative regulator of NF-*κ*B signaling (74). Consistent with its inflammatory role, mouse macrophages lacking miRNA-155 exhibit reduced responsiveness to LPS. Collectively, these findings establish miRNA-155 as a central regulator of pro-inflammatory macrophage programs. In addition, miRNA-155 suppresses anti-inflammatory macrophage programs by targeting components of the IL-4 and IL-13 pathways, including IL13RA and C/EBP (65, 75). Conversely, phenotypic changes toward anti-inflammatory and reparative macrophage programs are influenced by transcription factors such as STAT3, STAT6, IRF3, IRF4, peroxisome proliferator–activated receptor-*γ* (PPAR*γ*), and SMAD2, all of which are directly targeted by at least one miRNA (76).

Let-4c emerges as a miRNA closely associated with immunoregulatory macrophage activation. Overexpression of Let-4c enhances markers and functions linked to reparative programs, including efferocytosis, while concurrently suppressing pro-inflammatory gene expression. Mechanistically, Let-4c likely acts through regulation of CCAAT/enhancer-binding protein-*δ* (C/EBP-*δ*), a transcription factor that promotes LPS-driven inflammatory responses, as Let-4c reduces both mRNA and protein levels of C/EBP-*δ* (77).

## Regulation of macrophage function and foam cell formation by non-coding RNAs in atherosclerosis

Atherosclerosis is a multifactorial disorder encompassing numerous interrelated mechanisms, including dysregulated lipid and cholesterol metabolism, vascular smooth muscle cell (VSMC) migration, aberrant macrophage function, defective apoptosis, chronic inflammation, and fibrotic remodeling. Importantly, each of these processes is susceptible to regulation by genetic and epigenetic mechanisms, including noncoding RNAs and their complex interactions with cellular signaling networks (78). Indeed, multiple lncRNAs and miRNAs have been identified as either atheroprotective or atherogenic regulators (79, 80).

Elevated circulating LDL levels and impaired reverse cholesterol transport (RCT) mediated by high-density lipoprotein (HDL) are well-established risk factors for atherosclerosis (81, 82). Accumulation of oxidized LDL within the arterial wall activates inflammatory signaling pathways in endothelial cells, promoting monocyte recruitment, macrophage differentiation, and foam cell formation, ultimately driving plaque growth and instability (83). Therapeutic strategies that target systemic cholesterol metabolism can therefore attenuate immune cell activation and vascular inflammation at early stages of disease, a principle exemplified by statins among the most effective agents for reducing cardiovascular risk (84).

Beyond classical lipid-modifying therapies, emerging evidence highlights RNA-based regulatory mechanisms as critical modulators of cholesterol homeostasis and atherosclerosis progression. Several lncRNAs have emerged as important regulators of cholesterol homeostasis at both the systemic and macrophage levels. The lncRNA LASER (Lipid Associated Single Nucleotide Polymorphism Gene Region) increases circulating cholesterol levels by modulating hepatic LDL receptor turnover *in vitro* and *in vivo*. Mechanistically, LASER binds to lysine-specific demethylase 1 (LSD1), reducing H3K4 demethylation at the hepatocyte nuclear factor-1*α* (HNF-1*α*) promoter and thereby enhancing expression of proprotein convertase subtilisin/kexin type 9 (PCSK9). Increased PCSK9 promotes degradation of LDL receptors (LDLRs), limiting hepatic LDL uptake and elevating plasma LDL levels, thereby facilitating lipid accumulation within the vascular wall and promoting atherogenesis. Notably, LASER expression increases in statin-treated patients as part of a cholesterol-mediated feedback loop, suggesting that LASER induction may partially counteract statin efficacy. Targeting LASER therefore represents a potential strategy to enhance the effects of lipid-lowering therapies and further reduce cardiovascular risk (85).

Additional lncRNAs contribute to cholesterol regulation through liver- and macrophage-specific mechanisms. LeXis (Liver-expressed LXR-induced sequence) attenuates cholesterol biosynthesis by binding the ribonucleoprotein RALY in hepatocytes, thereby limiting RALY-mediated activation of cholesterol biosynthetic genes and reducing circulating cholesterol levels in murine models (86). In contrast, MeXis (Macrophage-expressed LXR-induced sequence) exerts atheroprotective effects within macrophages by guiding the transcriptional coactivator DDX17 to ABCA1 enhancers, thereby promoting cholesterol efflux and RCT (87). ABCA1, also known as cholesterol efflux regulatory protein (CERP), is a key mediator of HDL formation and macrophage lipid homeostasis (88).

miRNAs also play central roles in cholesterol metabolism and foam cell formation. Among these, miR-122 constitutes approximately 70% of hepatic miRNA expression and is a major regulator of systemic lipid metabolism (89). Antagonism of miR-122 using antagomirs reduces plasma cholesterol levels in mice (90). In addition, several other miRNAs have been predicted to regulate circulating cholesterol levels, although their precise molecular mechanisms remain incompletely characterized (91).

In macrophages, cholesterol influx, esterification, and efflux collectively determine intracellular lipid burden and foam cell stability (92). Several noncoding RNAs, predominantly miRNAs, have been implicated in these processes (93). Many of these miRNAs directly target ABCA1, thereby impairing cholesterol efflux and promoting oxidized LDL accumulation. These include miR-144-3p, miR-33, miR-19b, miR-101, miR-302a, miR-20, miR-26, miR-27, and miR-758 (94–103).

Lipid uptake pathways are also modulated by noncoding RNAs that regulate scavenger receptor expression. For example, the lncRNA MALAT1 mediates the oxidized LDL–induced increase in CD36 expression (104), also known as fatty acid translocase (FAT), which facilitates long-chain fatty acid uptake (105). NF-*κ*B signaling induces MALAT1 following oxidized LDL exposure, which in turn enhances lipid internalization in macrophages (104). Several miRNAs similarly influence lipid uptake, with miR-382-5p and miR-19a promoting this process, while miR-202-3p, miR-181a, miR-135a, miR-98, miR-150, miR-146b-5p, and miR-221-3p attenuating lipid accumulation (106), although many of these findings await in depth validation *in vivo*.

Beyond lipid handling, noncoding RNAs can influence foam cell formation by regulating monocyte recruitment and macrophage behavior independently of polarization status. The lncRNA NEXN-AS1 (Nexilin F-actin Binding Protein Antisense RNA 1) suppresses monocyte recruitment and foam cell formation by inhibiting the TLR4/NF-*κ*B pathway and modulating expression of the actin-binding protein NEXN, which regulates cell adhesion and migration. NEXN-AS1 expression decreases in human atherosclerotic arteries and exerts atheroprotective effects in murine models (107).

**Figure 2:**
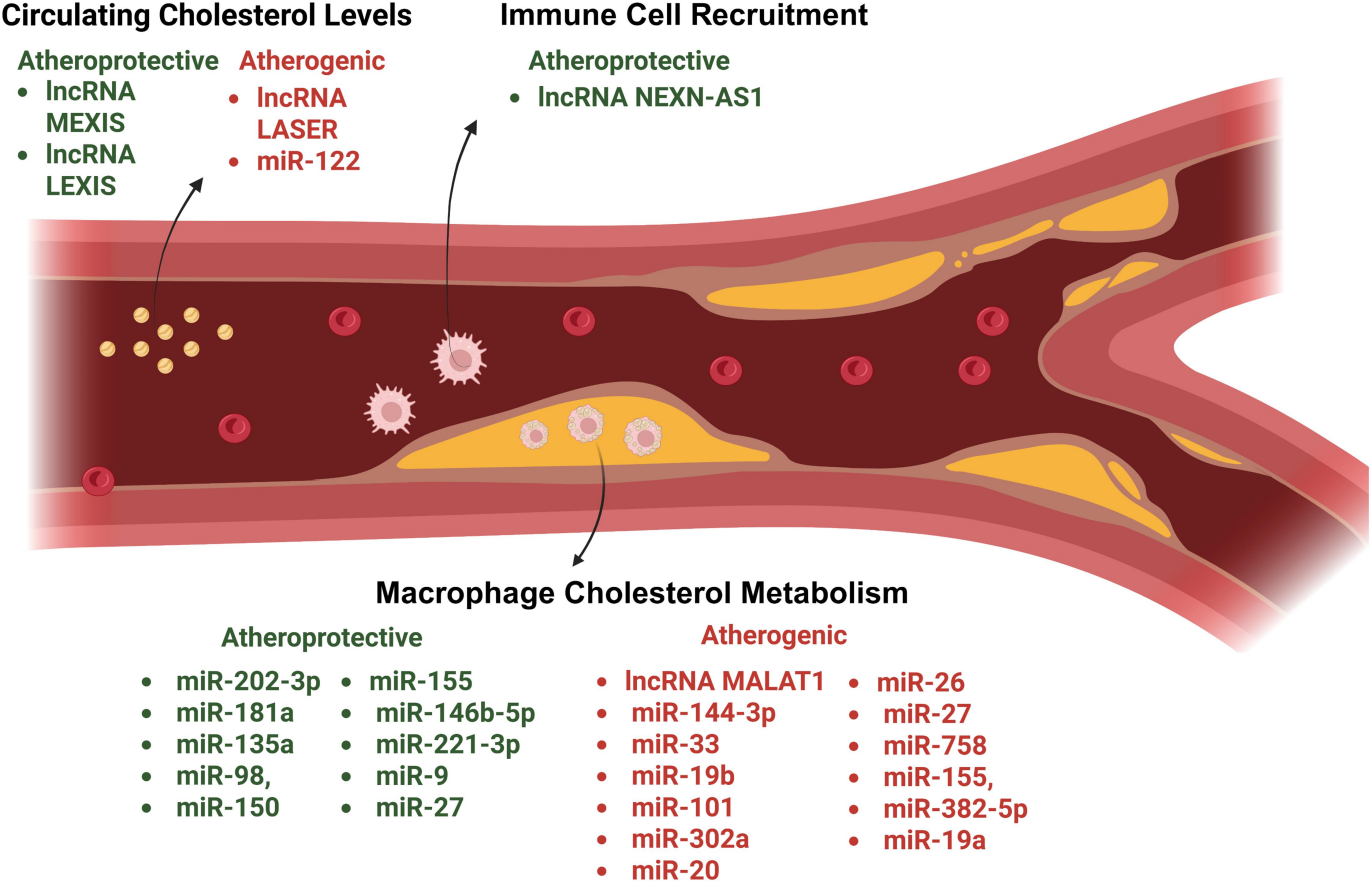
Noncoding RNA regulation of atherosclerosis-relevant processes. Schematic illustrating the roles of lncRNAs and microRNAs in circulating cholesterol levels, immune cell recruitment, and macrophage cholesterol metabolism during atherosclerosis. Atheroprotective and atherogenic noncoding RNAs are highlighted for each process, reflecting their opposing effects on lipid handling, inflammatory cell trafficking, and foam cell formation within the arterial wall.

Although macrophage activation state influences foam cell formation, with proinflammatory macrophages generally associated with disease progression and anti-inflammatory macrophages with regression, both phenotypes can contribute to lipid uptake under specific conditions. Accordingly, noncoding RNAs that regulate macrophage activation may indirectly modulate foam cell formation, while others act directly on lipid metabolic pathways. MiR-155 exemplifies this complexity, as it has been reported to exert atheroprotective effects in early disease stages but promote inflammation and plaque progression in advanced lesions (108). Figure 2 summarizes noncoding RNAs involved in macrophage recruitment and cholesterol metabolism, while Table 1 provides an overview of noncoding RNAs regulating cholesterol homeostasis and macrophage lipid metabolism in atherosclerosis.

**Table 1:** Major macrophage-associated noncoding RNAs in atherosclerosis and chronic kidney disease. This table summarizes representative long noncoding RNAs (lncRNAs) and microRNAs (miRNAs) implicated in the regulation of macrophage function across atherosclerosis and chronic kidney disease (CKD). Noncoding RNAs are categorized according to disease context, primary biological function, molecular targets or signaling pathways, and their overall effects on disease progression. The listed ncRNAs regulate key macrophage-associated processes including inflammatory activation, macrophage polarization, phagocytosis, chemotaxis, cholesterol homeostasis, foam-cell formation, tissue repair, and fibrosis. The table highlights both shared regulatory pathways operating across cardio-kidney-metabolic diseases and disease-specific mechanisms that contribute to vascular inflammation, atherosclerotic plaque progression, renal injury, and fibrotic remodeling. Together, these molecules represent potential biomarkers and therapeutic targets for precision medicine approaches aimed at modulating macrophage-driven pathology in cardiovascular and renal disease.

ncRNA	Disease Context	Main Function	Molecular Target/Mechanism	Effect
FENDRR	Shared	Pro-inflammatory macrophage activation	STAT1-associated signaling	Promotes inflammatory phenotype
LINC01010	Shared	Macrophage differentiation and phagocytosis	NF-*κ*B pathway	Supports macrophage function
miR-23	Shared/CKD	Anti-inflammatory	IRF1 inhibition	Suppresses pro-inflammatory macrophages
miR-146b	Shared/CKD	Anti-inflammatory	IRF5 inhibition	Limits inflammatory activation
miR-155	Shared	Pro-inflammatory	SOCS1/STAT1, BCL6	Promotes inflammatory macrophage programs
Let-7c	Shared	Reparative macrophage activation	C/EBP-*δ* inhibition	Enhances anti-inflammatory functions
MeXis	Atherosclerosis	Cholesterol efflux	ABCA1 activation via DDX17	Atheroprotective
LeXis	Atherosclerosis	Cholesterol homeostasis	RALY inhibition	Lowers cholesterol
LASER	Atherosclerosis	Cholesterol metabolism	PCSK9 induction	Increases LDL levels
MALAT1	Atherosclerosis	Lipid uptake	CD36 induction	Promotes foam-cell formation
NEXN-AS1	Atherosclerosis	Monocyte recruitment	TLR4/NF-κB inhibition	Atheroprotective
miR-33	Atherosclerosis	Cholesterol efflux	ABCA1 repression	Promotes foam-cell formation
miR-144-3p	Atherosclerosis	Cholesterol efflux	ABCA1 repression	Atherogenic
miR-9	Atherosclerosis	Cholesterol esterification	ACAT1 inhibition	Reduces foam-cell formation
SPANXA2-OT1	Atherosclerosis	Macrophage chemotaxis	miR-338/IL-8 axis	Enhances inflammation
lnc-FAM164A1	Atherosclerosis/Inflammation	Cytokine production	ACLY-NF-κB pathway	Promotes inflammation
lnc-TSI	CKD	Anti-fibrotic	Smad3 inhibition	Reduces fibrosis
miR-21	CKD	Pro-fibrotic	SMAD7 repression	Enhances fibrosis
miR-433	CKD	Pro-fibrotic	TGF-*β*/Smad3 amplification	Promotes fibrosis
miR-146a	CKD	Anti-inflammatory	SMAD4/TLR signaling regulation	Protective in kidney injury

## Noncoding RNAs in cholesterol esterification

Cholesterol esterification is a major determinant of cholesterol storage within foam cells. Acetyl-CoA acetyltransferases (ACATs) catalyze the conversion of free cholesterol into cholesterol esters in the endoplasmic reticulum (109). Although a relatively few noncoding RNAs have been directly linked to this process, emerging evidence supports the therapeutic potential of targeting specific miRNAs to reduce ACAT activity and foam cell formation.

MiR-9, extensively studied in oncological contexts, has been predicted to target the 3′ untranslated region of human *ACAT1* mRNA. Primary miR-9 transcripts are highly expressed in THP-1–derived macrophages, and miR-9 levels are inversely correlated with *ACAT1* expression. While miR-9 reduces ACAT1 protein levels without affecting mRNA abundance in murine cells suggesting translational repression miR-9 mimics significantly decrease foam cell formation in THP-1 macrophages exposed to oxidized LDL (110).

In addition to its role in cholesterol efflux, miR-27 also participates in cholesterol esterification. Both miR-27a and miR-27b regulate lipid metabolism–related genes and are predicted to target *ACAT1*127. Sequence alignment demonstrates strong complementarity between the *ACAT1* 3′ UTR and mature miR-27a/b sequences. Treatment of THP-1–derived foam cells with miR-27 mimics resulted in modest reductions in *ACAT1* mRNA but pronounced decreases in ACAT1 protein levels (111, 112).

## SPANXA2-OT1 and macrophage-driven atherogenesis

**Figure 3:**
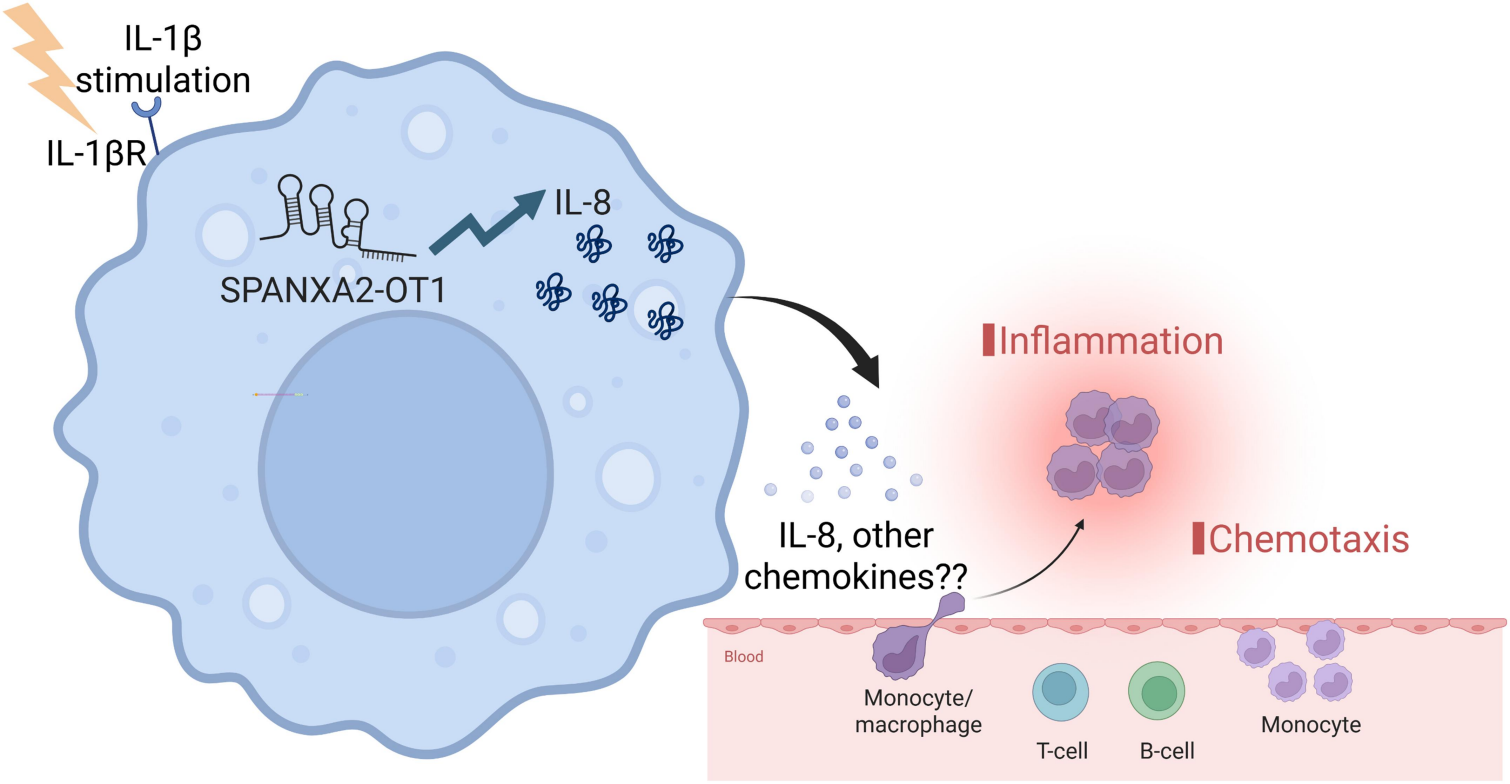
SPANXA2-OT1 mediates macrophage chemotaxis in atherosclerosis. IL-1β stimulation via IL-1β receptor (IL-1βR) induces the expression of the long noncoding RNA SPANXA2-OT1, which in turn promotes the production of IL-8 and potentially other chemokines. The increased secretion of these inflammatory mediators drives chemotaxis of monocytes/macrophages and enhances local inflammation. Modified from Jha et al., JCI Insight (23).

We recently identified the long noncoding RNA SPANXA2-OT1 as a primate-specific regulator of inflammation in coronary artery disease (CAD) that directly impacts macrophage function (23). Using integrative transcriptomic analyses in human cohorts, we found that the expression of SPANXA2-OT1 increases in peripheral immune cells from individuals with CAD compared with controls, motivating mechanistic studies in primary human macrophages (Figure 3). Functional studies demonstrated that SPANXA2-OT1 is induced by inflammatory stimulation and predominantly localizes to the cytoplasm. SPANXA2-OT1 regulates chemokines involved in macrophage recruitment, including IL-8, a key mediator of monocyte chemotaxis and inflammatory signaling. Mechanistically, SPANXA2-OT1 acts as a molecular sponge for miR-338, relieving repression of IL-8 and thereby enhancing inflammatory chemokine output and macrophage migratory capacity. Loss of SPANXA2-OT1 activity either by CRISPR/Cas9 deletion of its functional domain or by genetic perturbation decreased macrophage chemotaxis and altered inflammatory and chemokine gene signatures, establishing a causal role for SPANXA2-OT1 in macrophage-mediated inflammatory processes relevant to atherogenesis. These findings position SPANXA2-OT1 as a novel human-specific regulator of macrophage chemotaxis and inflammation in CAD, with potential utility as both a biomarker and therapeutic target in atherosclerotic cardiovascular disease (23).

## The lnc-FAM164A1-ACL*Y* axis promotes pro-inflammatory responses in human primary macrophages

In our recent study, microarray analysis of lipopolysaccharides (LPS)-stimulated human primary macrophages identified *lnc-FAM164A1* as a new regulator of inflammatory disease. Loss-of-function and gain-of-function experiments revealed *lnc-FAM164A1* regulates the LPS-triggered induction of the pro-inflammatory molecules CCL2/MCP-1 and TNF-*α* via the NF-*κ*B pathway. In humanized mice, LPS induced *lnc-FAM164A1* in blood leukocytes and spleen. Proteomics of RNA pulldown samples identified ATP-citrate synthase (ACLY) as a *lnc-FAM164A1*-associated protein, which regulates CCL2 and TNF-*α*. These novel findings provided a molecular basis for the potential development of *lnc-FAM164A1*-targeted therapeutics against macrophage-mediated inflammatory vascular diseases (113).

## Noncoding RNAs mediated macrophage function in CKD

CKD is a multisystemic disorder, most commonly observed in patients with diabetes or hypertension. It is characterized by a gradual decline in kidney function or the presence of kidney damage markers for at least three months (114). Given the kidneys' central role in regulating blood pressure, electrolyte balance, and metabolic homeostasis, CKD significantly increases cardiovascular risk and is closely linked to cardio-kidney-metabolic disorders (35, 115). Macrophages are pivotal in CKD pathophysiology (21). Evidence suggests that proinflammatory macrophages dominate early inflammatory infiltration in the kidney, whereas anti-inflammatory macrophages are prevalent during later, fibrotic stages (116). Consequently, therapeutic strategies targeting macrophage-mediated renal fibrosis hold considerable promise. Suppressing proinflammatory macrophage responses while preserving reparative macrophage functions may promote tissue repair and slow disease progression (117). These observations underscore the importance of macrophage function and the noncoding RNAs that regulate it in CKD pathogenesis.

Consistent with this, numerous noncoding RNAs, whether directly linked to macrophage function or not, have been implicated in CKD regulation. In a classic mouse model of renal fibrosis, unilateral ureteral obstruction (UUO), 625 lncRNAs were found to be upregulated in urine. Among these, 19 contained several conserved Smad3-binding motifs (118). Smad3, a key mediator of pro-fibrotic signaling, drives transcription of multiple lncRNAs and miRNAs and is central to the TGF-*β*/Smad signaling pathway, which mediates macrophage driven fibrosis in later CKD stages (119–121).

TGF-*β*, a well-established central regulator of renal fibrosis, induces promoters of lncRNAs with multiple Smad3-binding sequences. One such lncRNA, lnc-TSI, binds to the MH2 domain of Smad3, blocking its interaction with the TGF-*β* receptor and thereby inhibiting downstream fibrotic signaling. Delivery of human lnc-TSI into UUO mice significantly reduced renal fibrogenesis, highlighting its therapeutic potential (122).

miRNAs also modulate fibrosis in CKD, although many have been studied primarily as biomarkers. For example, TGF-*β* induces miR-433, promoting renal fibrosis *in vitro*; its overexpression enhances TGF-*β*1–driven fibrotic signaling, whereas its silencing attenuates this response (123). Other miRNAs, including miR-146a, miR-203, and miR-548d, regulate SMAD4, a transcriptional activator of TGF-*β*. Suppression of these miRNAs enhances SMAD4 expression and amplifies TGF-*β* signaling (124, 125). Additionally, miR-21 targets SMAD7, a negative regulator of TGF-*β*/Smad3 signaling, contributing to renal fibrosis (126). Beyond fibrosis, several noncoding RNAs directly regulate macrophage activation and inflammatory responses relevant to CKD progression. For example, miR-23 suppresses pro-inflammatory macrophage polarization through inhibition of the IRF1 pathway, whereas miR-146 family members attenuate inflammatory macrophage activation by targeting IRF5-dependent signaling (70, 71, 124). Conversely, miR-155 promotes pro-inflammatory macrophage programs through the SOCS1/STAT1 axis and amplifies inflammatory cytokine production (65, 69). Notably, these ncRNAs also regulate macrophage activation states in atherosclerosis, suggesting the existence of shared inflammatory regulatory networks across cardio-kidney-metabolic diseases. However, downstream pathological consequences differ between disease settings. In atherosclerosis, macrophage-associated ncRNAs frequently control lipid uptake, cholesterol efflux, foam cell formation, and plaque remodeling (78, 92), whereas in CKD they more commonly influence renal inflammation, TGF-*β*/Smad-driven fibrogenesis, and tissue repair processes (119, 120). These observations support the concept that common ncRNA-dependent macrophage programs operate across multiple chronic inflammatory diseases while driving distinct organ-specific outcomes.

**Figure 4:**
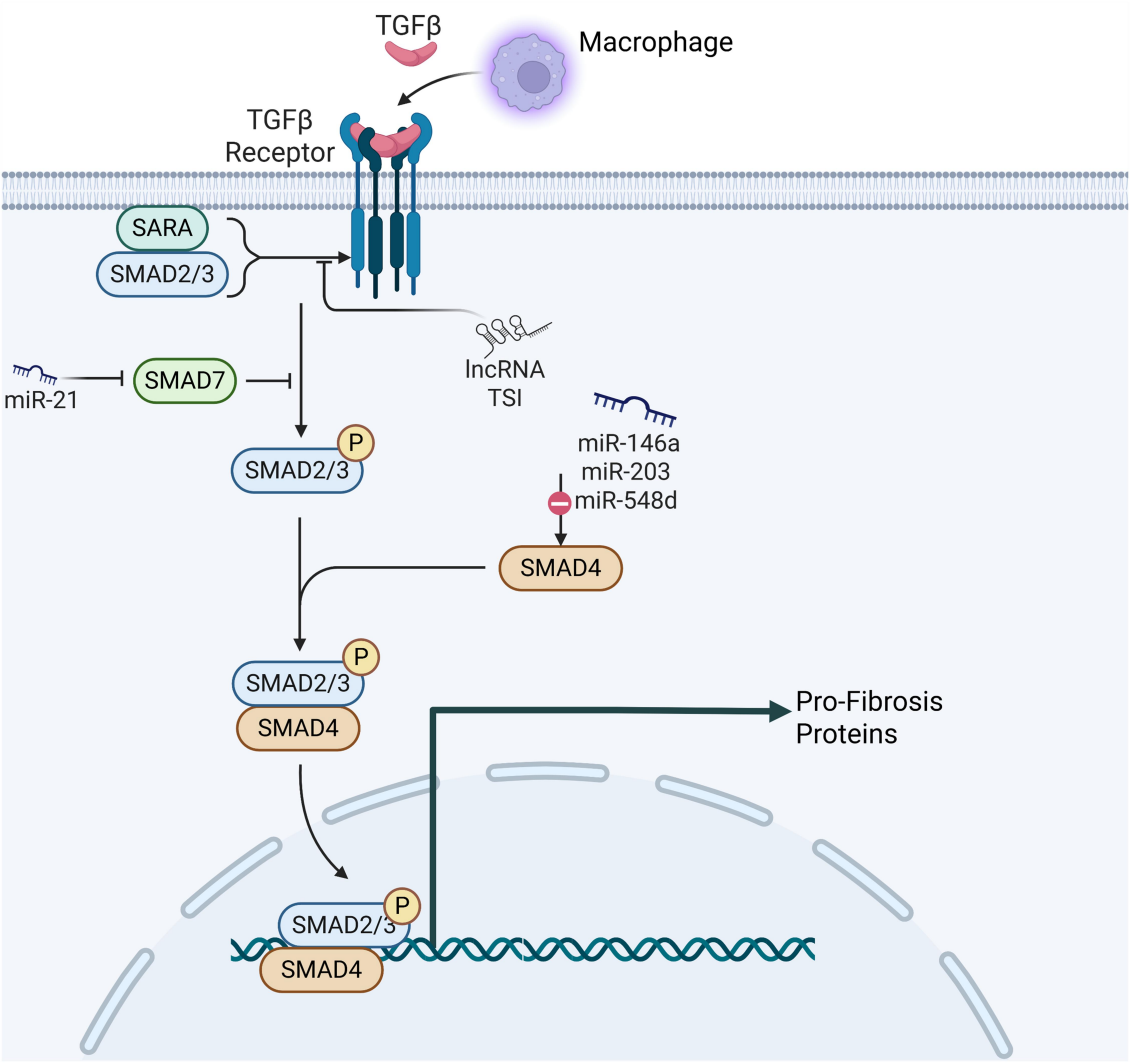
Noncoding RNAs acting in fibrosis pathway regulation in chronic kidney disease. Schematic depicting TGF-*β* signaling, where ligand released from macrophage binds to TGF-*β* receptors induces SMAD2/3 phosphorylation and complex formation with SMAD4, followed by nuclear translocation and transcription of pro-fibrotic genes. The pathway is fine-tuned by noncoding RNAs, including inhibitory SMAD7 and miR-21, as well as lncRNA TSI and miRNAs (miR-146a, miR-203, miR-548d) that modulate SMAD activation and SMAD4 availability.

Notably, several macrophage-associated ncRNAs, including miR-23, miR-146, and miR-155, regulate inflammatory macrophage programs in both vascular and renal disease settings, suggesting the existence of conserved regulatory networks across the cardio-kidney-metabolic axis. In contrast, disease-specific ncRNA functions emerge through regulation of lipid metabolism in atherosclerosis and fibrotic remodeling in CKD. Figure 4 illustrates key noncoding RNAs involved in fibrosis pathway regulation in CKD.

## Shared macrophage-associated noncoding RNA pathways across atherosclerosis and CKD

Although atherosclerosis and CKD manifest in distinct organs, both diseases are characterized by chronic inflammation, sustained macrophage activation, and maladaptive tissue remodeling. Recent evidence suggests that macrophage dysfunction contributes to these pathological processes through shared regulatory networks that govern macrophage heterogeneity, inflammatory signaling, and disease progression (19, 21, 39, 43, 49). Macrophages serve as key cellular effectors in both conditions, integrating environmental and metabolic cues to drive diverse phenotypic states that can either promote tissue injury or support repair (20, 43, 44, 50, 51).

Several ncRNAs regulate common macrophage activation pathways that are implicated in both vascular and renal disease. For example, miR-23 suppresses pro-inflammatory macrophage polarization through inhibition of the IRF1 pathway, whereas members of the miR-146 family restrain inflammatory activation by targeting IRF5-dependent signaling (70, 71). In contrast, miR-155 enhances pro-inflammatory macrophage programs through modulation of the SOCS1/STAT1 axis and suppression of anti-inflammatory signaling pathways (65, 69, 74, 75). These observations suggest that multiple ncRNAs converge on conserved transcriptional networks that govern macrophage inflammatory responses across different disease settings.

Despite these shared mechanisms, disease-specific downstream consequences are shaped by local tissue environments. In atherosclerosis, macrophage-associated ncRNAs frequently regulate lipid uptake, cholesterol efflux, foam cell formation, and plaque remodeling through targets such as ABCA1, CD36, and ACAT1 (87, 94, 97, 104, 110). In CKD, ncRNAs more commonly influence TGF-*β*/Smad signaling, macrophage-driven fibrosis, and tissue repair processes that determine progression toward chronic renal dysfunction (119, 120, 122, 123). Thus, while common inflammatory macrophage programs operate across both diseases, they ultimately contribute to distinct pathological outcomes.

Taken together, these findings support the concept of a cardio-kidney-metabolic disease continuum in which ncRNA-mediated regulation of macrophage function represents a shared pathogenic axis linking atherosclerosis and CKD. A deeper understanding of these common regulatory networks may reveal therapeutic targets capable of simultaneously attenuating vascular inflammation, renal injury, and macrophage-driven disease progression across multiple organ systems.

## Conclusions and future perspectives

Noncoding RNAs, including both lncRNAs and miRNAs, have emerged as central regulators of macrophage function in cardio-kidney-metabolic diseases such as atherosclerosis and CKD. By modulating macrophage function, lipid metabolism, inflammatory signaling, and fibrotic pathways, these molecules orchestrate key cellular processes that drive disease onset, progression, and tissue remodeling. Studies to date, including our own identification of SPANXA2-OT1, highlight the complexity and therapeutic potential of these regulatory networks.

Despite significant advances, several critical gaps remain. First, while numerous noncoding RNAs have been identified *in vitro* and in preclinical models, their precise roles in human disease contexts remain incompletely understood, and relatively few candidate ncRNAs have undergone rigorous validation in large clinical cohorts. Many lncRNAs and miRNAs likely exert context-dependent, cell type-specific, and disease stage-specific effects that require further investigation. Second, the interplay between different noncoding RNAs and their combined influence on macrophage behavior remains largely unexplored. For example, lncRNAs can act as molecular sponges for miRNAs, creating intricate regulatory circuits that are just beginning to be characterized. Third, although noncoding RNAs represent promising therapeutic targets, challenges in delivery, stability, and off-target effects need to be addressed before clinical translation can be realized.

Future research should therefore focus on several key directions. Integrating single-cell and spatial transcriptomics with functional assays will enable a more precise mapping of noncoding RNA-mediated regulation in distinct macrophage subsets across different stages of disease. High-throughput CRISPR-based perturbation screens and advanced *in vivo* models can uncover causal relationships between specific noncoding RNAs and pathogenic processes. Furthermore, elucidating shared and divergent noncoding RNA pathways between atherosclerosis and CKD may reveal novel targets for interventions that simultaneously ameliorate vascular and renal complications.

Despite their considerable therapeutic promise, several challenges currently limit the clinical translation of ncRNA-based therapies. Many ncRNAs exert cell type-specific and context-dependent effects, raising concerns regarding unintended consequences of systemic modulation. In addition, therapeutic RNAs may exhibit limited stability, rapid degradation, suboptimal tissue penetration, immune activation, and off-target effects. These challenges are particularly relevant for lncRNAs, whose mechanisms of action remain incompletely understood and whose sequence conservation across species is often limited. Nevertheless, recent advances in RNA therapeutics have provided promising solutions. Antisense oligonucleotides (ASOs), miRNA mimics, miRNA inhibitors, and small interfering RNA-based approaches offer opportunities for selective modulation of disease-associated ncRNAs. Improvements in oligonucleotide chemistry have enhanced molecular stability and target engagement, while lipid nanoparticle (LNP)-based delivery systems have significantly improved intracellular delivery efficiency and pharmacokinetic properties. Emerging macrophage-targeted delivery strategies, including nanoparticles engineered to recognize macrophage-enriched surface receptors within atherosclerotic plaques or injured kidneys, may further increase therapeutic specificity while minimizing systemic toxicity. In parallel, the development of robust biomarkers and pharmacodynamic readouts for ncRNA target engagement will be critical for patient stratification, dose optimization, and clinical translation. Together, these advances are bringing ncRNA-based precision therapeutics closer to clinical application for macrophage-driven cardiovascular and renal diseases. In summary, noncoding RNAs represent a critical layer of regulation in macrophage-driven cardio-kidney-metabolic disease. Continued mechanistic studies, combined with translational and therapeutic exploration, hold the promise of advancing precision medicine approaches aimed at modulating macrophage function and mitigating the burden of atherosclerosis and CKD.
